# Complex Role of Capsaicin-Sensitive Afferents in the Collagen Antibody-Induced Autoimmune Arthritis of the Mouse

**DOI:** 10.1038/s41598-018-34005-6

**Published:** 2018-10-29

**Authors:** Éva Borbély, Tamás Kiss, Krisztina Szabadfi, Erika Pintér, János Szolcsányi, Zsuzsanna Helyes, Bálint Botz

**Affiliations:** 1 University of Pécs, Medical School, Department of Pharmacology and Pharmacotherapy, János Szentágothai Research Centre, Molecular Pharmacology Research Team & Centre for Neuroscience, Pécs, Hungary; 2 University of Pécs, Department of Experimental Zoology and Neurobiology, Pécs, Hungary; 3 University of Pécs, Medical School, Department of Radiology, Pécs, Hungary

## Abstract

Capsaicin-sensitive afferents have complex regulatory functions in the joints orchestrated via neuropeptides. This study aimed to determine their role in the collagen-antibody induced rheumatoid arthritis model. Capsaicin-sensitive nerves were defunctionalized by the capsaicin receptor agonist resiniferatoxin in C57Bl/6 mice. Arthritis was induced by the ArithroMab antibody cocktail and adjuvant. Arthritis was monitored by measuring body weight, joint edema by plethysmometry, arthritis severity by clinical scoring, mechanonociceptive threshold by plantar esthesiometry, thermonociceptive threshold by hot plate, cold tolerance by paw withdrawal latency from 0 °C water. Grasping ability was determined by the wire-grid grip test. Bone structure was evaluated by *in vivo* micro-CT and histology. Arthritic animals developed a modest joint edema, mechanical and cold hyperalgesia, weight loss, and a diminished grasping function, while thermal hyperalgesia is absent in the model. Desensitised mice displayed reduced arthritis severity, edema, and mechanical hyperalgesia, however, cold hyperalgesia was significantly greater in this group. Arthritic controls displayed a transient decrease of bone volume and an increased porosity, while bone density and trabecularity increased in desensitised mice. The activation of capsaicin-sensitive afferents increases joint inflammation and mechanical hyperalgesia, but decreases cold allodynia. It also affects inflammatory bone structural changes by promoting bone resorption.

## Introduction

Rheumatoid arthritis (RA) is a chronic autouimmune inflammatory disease of the joints, constituting a global burden. RA is a public health problem due to its high prevalence, affecting 0.1–1% of the world population with significant regional differences^1^. In the last two decades the introduction of novel drugs, primarily biologics, improved the treatment of the immune-component of RA^2^. However, the analgesic therapeutic regime underwent only minimal change. The evidence of the complex interplay of neural and immune components in the development of RA has been proven by numerous clinical and experimental studies, but the mechanism of these interactions is still incompletely understood. Early experimental results^3^, and occassional clinical observations^4^ showed that local denervation is protective against joint inflammation. This highlighted the critical importance of innervation in the induction of arthritis.

Capsaicin-sensitive sensory afferents densely innervate the articular capsule and the synovium, hence their involvement in arthritic pain has been proposed relatively early on^5^. A hallmark feature of these nerve terminals is their dual nature: as classic afferents they participate in pain signaling towards the central nervous system and they also modulate the inflammatory reaction by acting as efferents by the release of sensory neuropeptides. A key feature of these nerve terminals is the expression of the Transient Receptor Potential Vanilloid 1 (TRPV1) capsaicin receptor, which is a non-selective cation channel. It is activated and sensitized not only by a plethora of exogenous irritants, but also by endogenous inflammatory mediators also upregulated during RA^6^. This includes noxious heat, protons, prostanoids, bradkyinin, TNF-α, and free radicals, but also phytochemicals like capsaicin, the pungent ingredient of chilli pepper, and its even more potent analogue resiniferatoxin (RTX)^5,7^. TRPV1 receptor activation results in the release of peptide mediators, some of which are proinflammatory, such as tachykinins, calcitonin gene-releated peptide. These in turn induce local hyperemia and the recruitment of inflammatory cells, which is called neurogenic inflammation^8^. However, simultaneously, anti-inflammatory mediators, like somatostatin are also released. The clinical importance of such peptide mediators is well established, and numerous studies have shown that RA and also osteoarthritis patients display altered levels of sensory neuropeptides in the synovial fluid and/or serum. Furthermore, recent studies also identified single nucleotide polymorphisms (SNP) responsible for this increased expression^9–15^. Large scale human studies have also discovered that certain SNPs of neuropeptide receptors increase the likelihood of symptomatic arthritis^16^. Sporadic clinical evidence also shows that anti-inflammatory neuropeptide mediators are able to delay the onset of RA, demonstrated by cases of rapidly developing RA immediately after the successful treatment of somatostatin-producing endocrine tumors^17^. Prior work has provided compelling, albeit in several cases conflicting evidence of the importance of both capsaicin-sensitive afferents, TRPV1 ion channels, and the divergent pro- and antiinflammatory role of their mediators using diverse models of joint inflammation mimicking different aspects of RA^18–21^.

The collagen-induced arthritis (CIA) is widely known as a gold standard murine model of RA, but due to its variable incidence, disease severity, and limited susceptibility of standard laboratory mouse strains using this model is often not feasible, and comparisons remain a challenge. The CIA model is characterized by a high titer of anti-type II collagen IgG autoantibodies, which is also a hallmark feature of RA. Passive transfer of the antibodies of CIA mice into healthy recipients can induce a more rapid, albeit transient and mild arthritis with 100% penetration in many mouse strains not readily susceptible to CIA, hence it is termed collagen-antibody-induced arthritis (CAIA). Unlike the original model, CAIA is mediated mainly via macrophages and neutrophils, without any involvement of the adaptive immunity^22,23^.

In the present study we aimed to characterize the role of capsaicin-sensitive afferents in the CAIA model of RA, which offers an unique way to interrogate the involvement of these nerve endings in anti-type II collagen antibody-driven joint inflammation. Furthermore, we also evaluated the performance of the model in the C57Bl/6 strain, which is widely used for interrogating mechanisms of pain and inflammation, but is not readily susceptible to other collagen-induced arthritis models.

## Results

### Decreased edema and arthritis severity in desensitized arthritic mice

**Figure 1:**
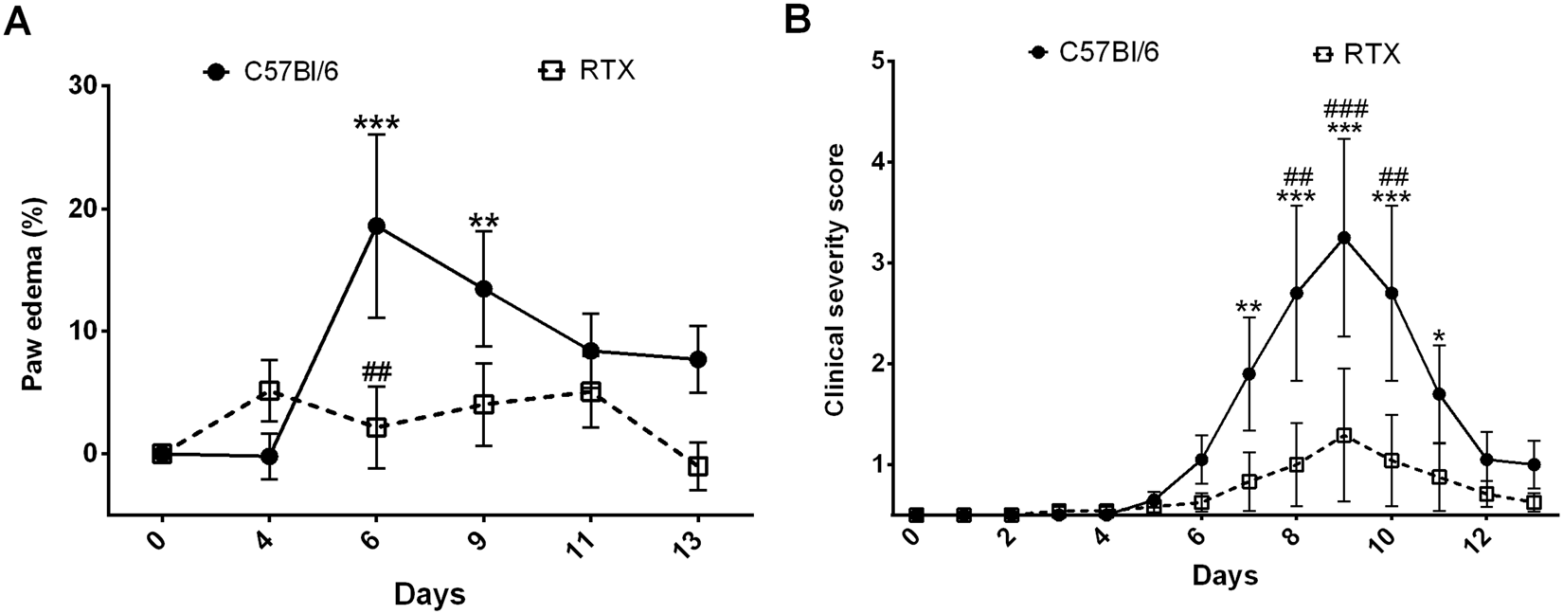
Changes of (**A**) hindpaw volume, (**B**) clinical arthritis severity score. Data points represent mean ± SEM. (n = 5–6/group, *p < 0.05, **p < 0.01, ***p < 0.001 vs. initial self control, ^##^p < 0.01, ^###^p < 0.001 vs. non-pretreated, two-way repeated measures ANOVA followed by Sidak’s post-test.)

Hindpaw edema and clinical arthritis severity scores remained unchanged in both groups until day 3, when the lipopolysaccharide booster injection was administered. In non-desensitized mice a modest, but significant 20% increase of hindlimb volume was observed, which peaked on day 6 (Fig. 1A). Arthritis severity scores reached their maximum on day 9 having the maximal values of 3–4 in non-pretreated mice, indicating moderate, but significant arthritic changes (Fig. 1B). In desensitized mice no significant edema formation, or clinical severity score increase could be observed.

### Diminished mechanical hyperalgesia, but increased cold allodynia in desensitized mice

**Figure 2:**
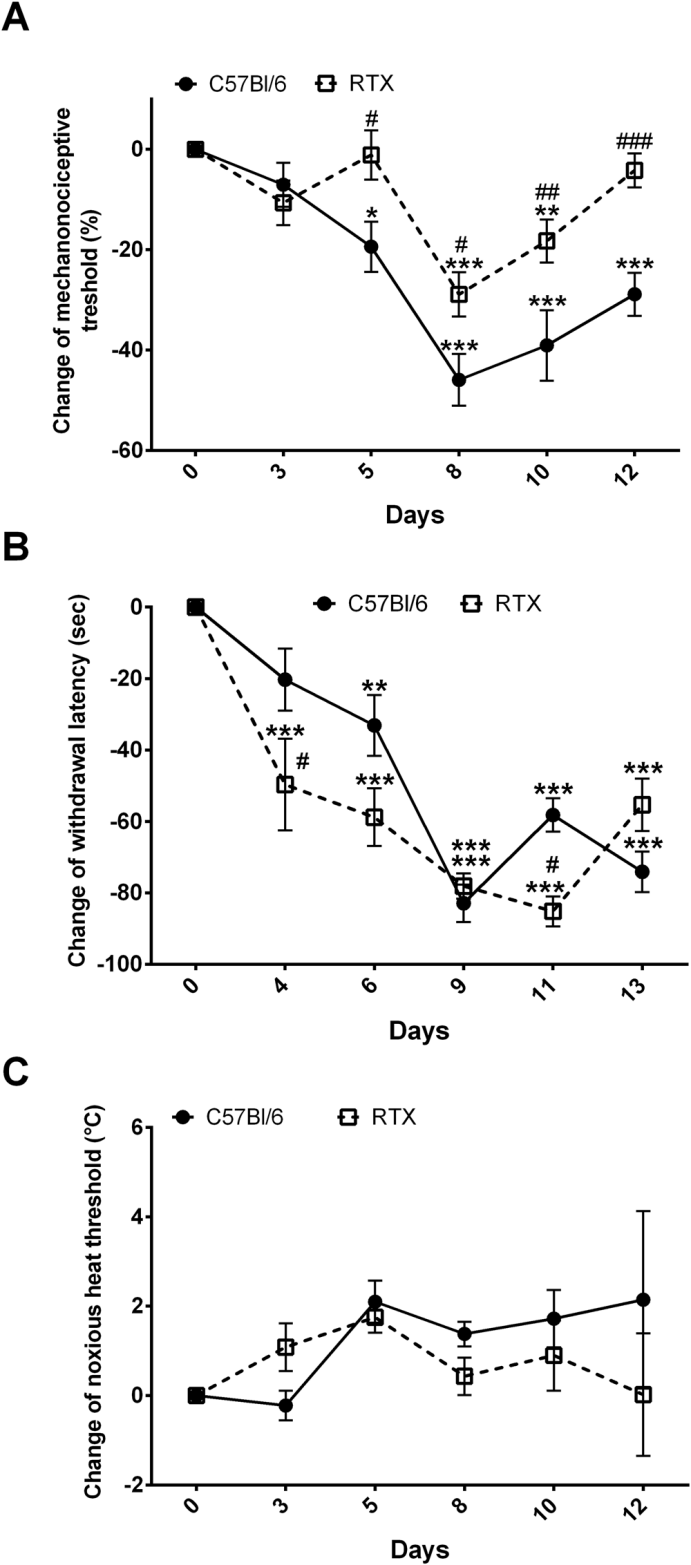
Changes of (**A**) mechanical hyperalgesia, (**B**) cold allodynia, (**C**) thermal hyperalgesia. Data points represent mean ± SEM. (n = 5–6/group, *p < 0.05, **p < 0.01, ***p < 0.001 vs. initial self-control, ^#^p < 0.05, ^##^p < 0.01, ^###^p < 0.001 vs. non-pretreated, two-way repeated measures ANOVA followed by Sidak’s post-test).

The baseline touch sensitivity threshold of the non-desensitized and RTX-desensitized groups were not significantly different. In non-desensitized mice a 45% of mechanonociceptive threshold drop was observed, peaking on day 8. In RTX-pretreated mice inflammatory mechanical hyperalgesia peaked at 30%, and remained significantly lower thereafter (Fig. 2A). Paw withdrawal latency from ice-cold water gradually decreased during the study in both groups, being significantly lower in desensitized animals on days 4 and 11 showing cold hyperalgesia in the model (Fig. 2B). Thermal hyperalgesia did not develop in the model in either group (Fig. 2C).

### Absent difference in weight loss and grasping ability in arthritic mice after sensory desensitization

**Figure 3:**
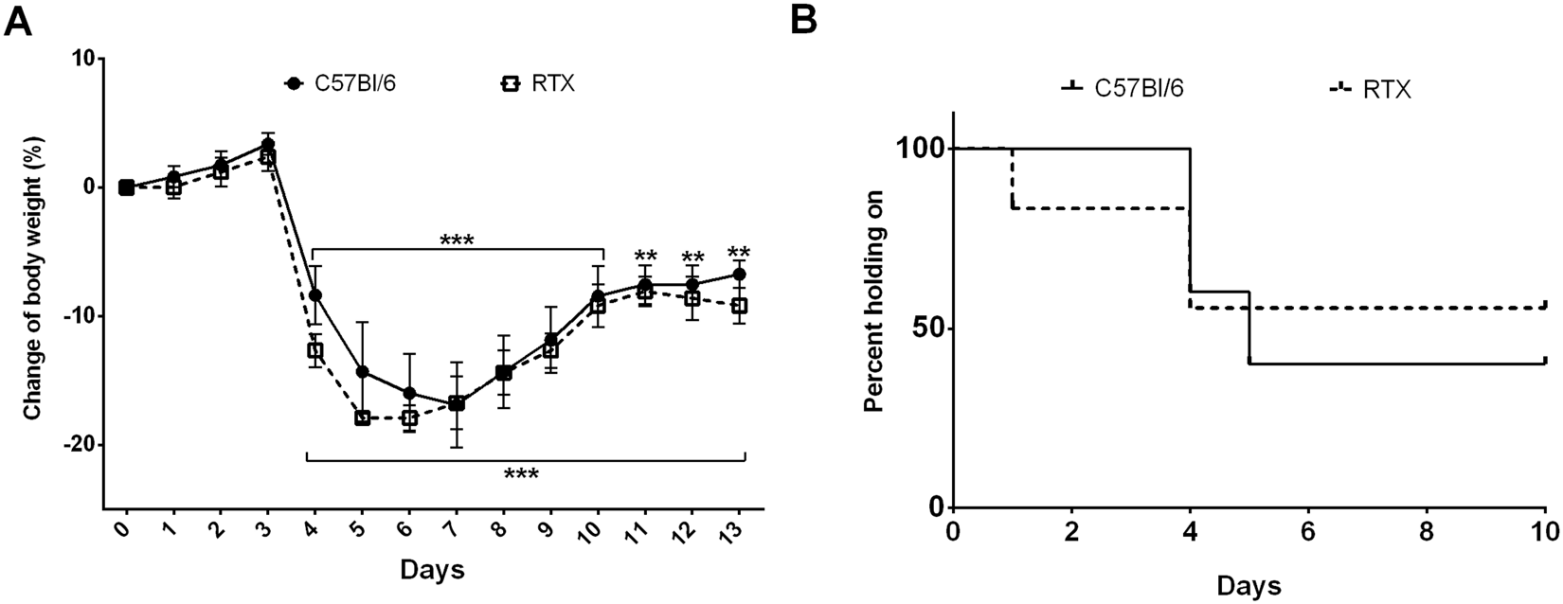
Changes of (**A**) body weight (two-way repeated measures ANOVA followed by Sidak’s post-test), (**B**) wire grid grasping ability (Mantel-Cox test). (n = 5–6/group, **p < 0.01, ***p < 0.001 vs. initial self-control).

A 20% weight loss developed rapidly upon arthritis induction in both desensitized and non-pretreated mice, peaking on days 6–7 of the study (Fig. 3A). Wire grid performance decreased steadily during the first half of study and by day 5 about 50% of the mice in both groups became unable to maintain their position on the wire mesh grid for the test period, however no significant difference could be observed (Fig. 3B). The grasping ability returned to normal in both groups after day 12 (Supplementary Fig. S1).

### Absent bone resorption, increased bone volume, but diminished bone pore formation in desensitized arthritic animals

**Figure 4:**
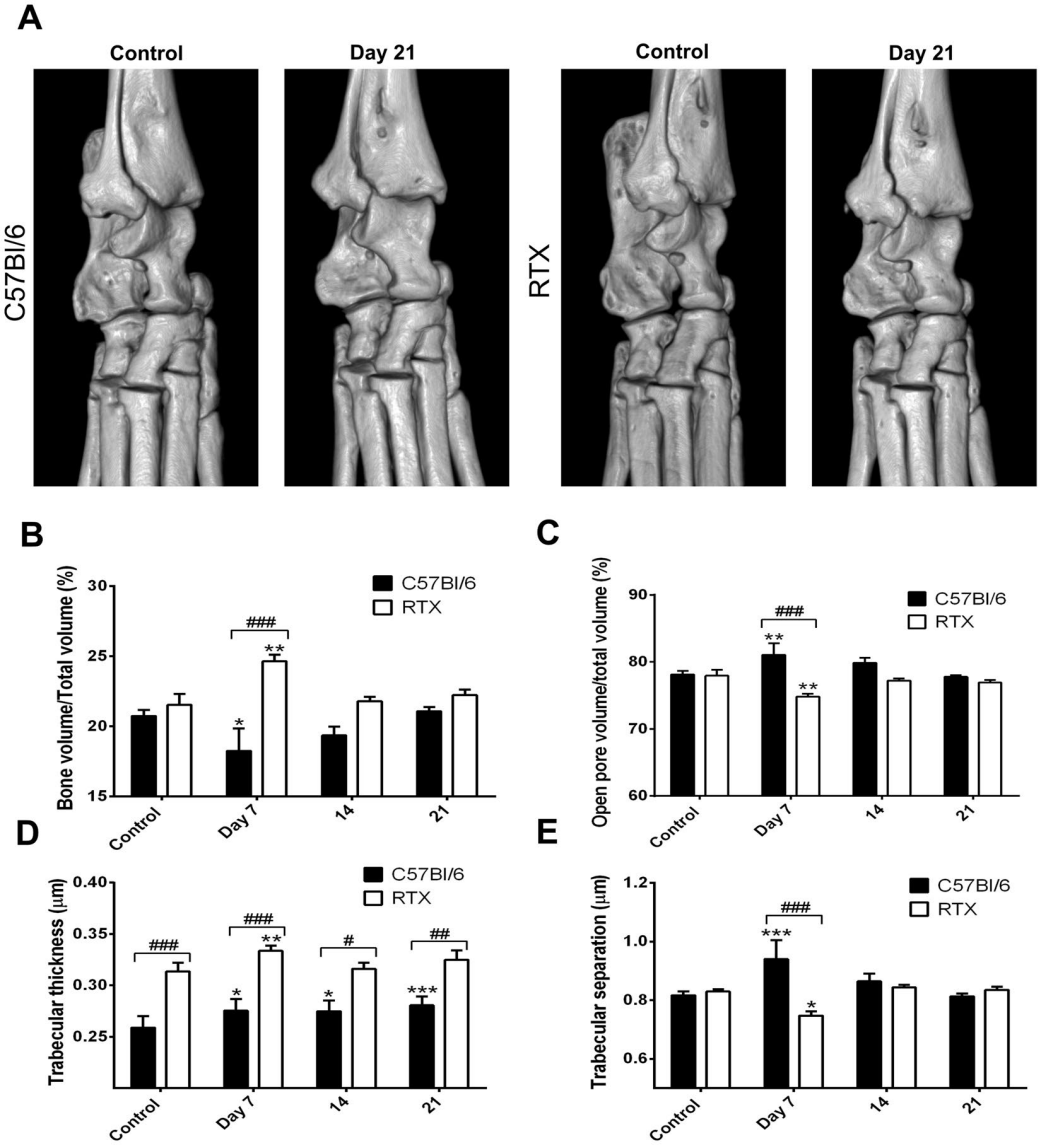
The changes of bone structural parameters throughout the study. (**A**) representative micro-CT images of the ankle joints. Changes of (**B**) bone mass, (**C**) percent volume of open pores, (**D**) mean thickness, (**E**) mean separation of bone trabecules. (n = 5–6/group, *p < 0.05, **p < 0.01, ***p < 0.001 vs. initial self control, ^#^p < 0.05, ^##^p < 0.01, ^###^p < 0.001 vs. non-pretreated, two-way repeated measures ANOVA followed by Sidak’s post-test).

No visually apparent bone erosions or osteophytes developed around the ankle joints during the study, reflecting the transient nature of the model (Fig. 4A). RTX-desensitization alone did not influence bone mass. However a significant 15% increase of the bone volume/total volume ratio could be observed in desensitized mice by day 7, while non-pretreated animals displayed a 12% decrease (Fig. 4B). The percentage of open pores increased significantly in non-pretreated, but not in desensitized mice by day 7, but this difference was not maintained for the rest of the study (Fig. 4C). On the other hand, bone trabecular thickness was found to be approximately 20% greater in RTX-pretreated mice, a difference which was consistently maintained throughout the study (Fig. 4D). Trabecular separation increased remarkably by 15% in non-pretreated mice, while decreasing by 10% after desensitization by day 7 (Fig. 4E).

### Retained integrity of the articular cartilage

**Figure 5:**
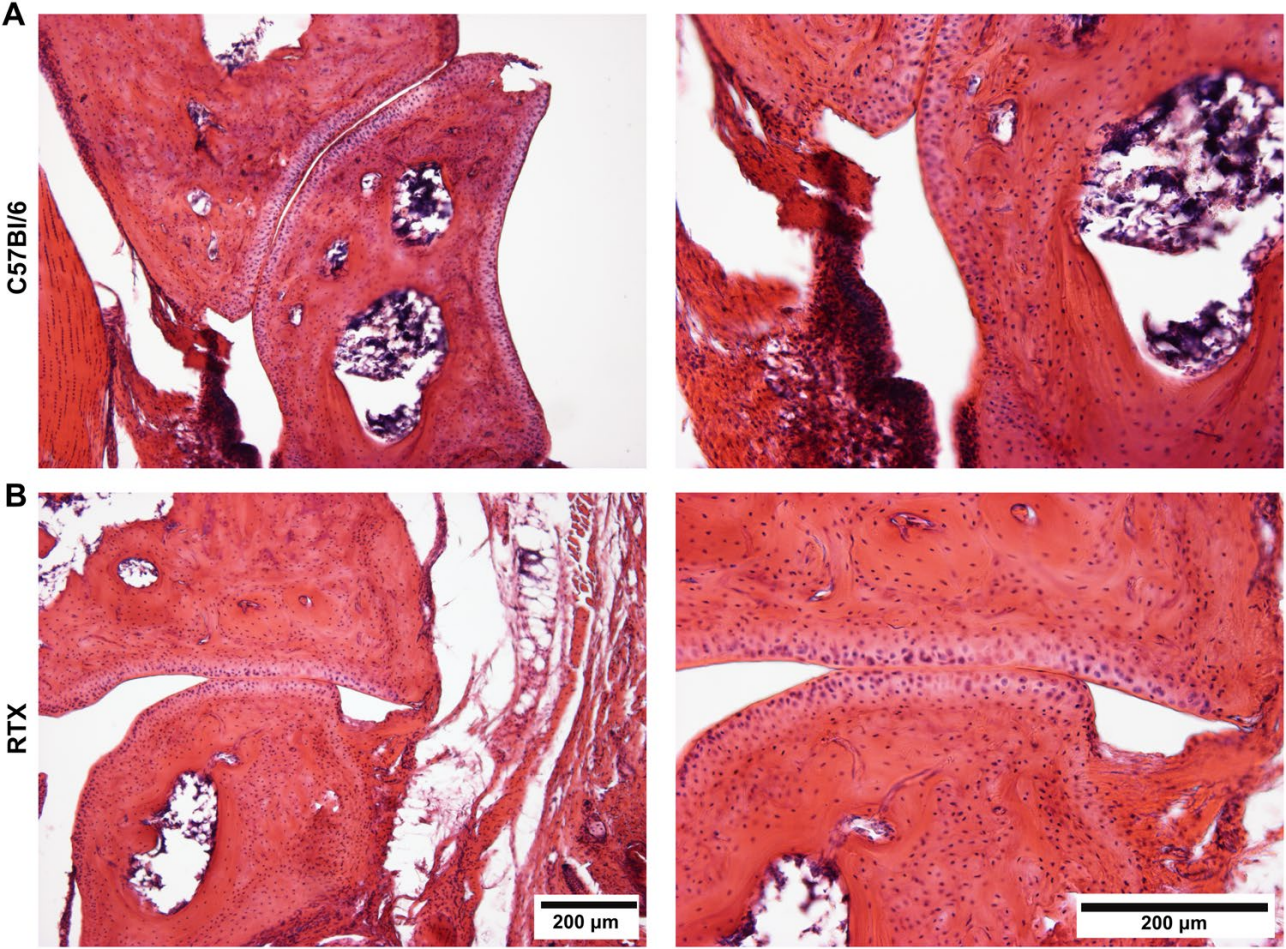
Representative histological slides of the tibiotarsal joints excised at the end of the experiment (day 21) stained with hematoxylin-eosin of (**A**) arthritic control, and (**B**) RTX-pretreated mice (100x and 200x magnification).

Examination of the histological slides made from the ankle joints obtained on day 21 demonstrated mild synovial proliferation in the tibiotarsal joints of both arthritic groups, but the articular cartilage remained intact (Fig. 5A,B).

## Discussion

Our experiment provides the first evidence for the involvement of capsaicin-sensitive sensory nerves in the CAIA model of autoantibody-induced joint inflammation. Desensitization of these afferents results in decreased edema-formation and clinical arthritis severity, along with diminished mechanical hyperalgesia and bone resorption, but leads to increased cold allodynia.

Since there is no completely relevant disease model of RA that could mimic all mechanisms and phases of the human condition, it is important to compare the results obtained in models of different arthritis-related mechanisms to conclude on the role of the capsaicin-sensitive peptidergic sensory nerves.

In previous studies using the CFA-induced arthritis model and the K/BxN serum-transfer model systemic or topical RTX-pretreatment increased arthritis severity, but diminished mechanical hyperalgesia^18,19,24^. In the more transient mast cell tryptase-induced knee inflammation model edema was similar, while spontaneous pain was diminished after desensitization^21^. The decreased mechanical hyperalgesia is in good agreement with these previous results, but in the CAIA-model the observed decreased inflammation severity and edema seem to contradict our earlier results. This likely reflects the different inflammatory pathways involved in these models. Unlike the CAIA model, the K/BxN model mediated by different autoantibodies (anti-glucose-6-phosphate isomerase) eliciting a rapidly occurring vascular leakage dependent on mast cells, neutrophils, and the Fc-gamma receptor III (FcγRIII)^22,25^. Therefore, the exact inflammatory signaling cascade occurs via distinct pathways, leading to different overall effects of the desensitization of capsaicin-sensitive afferents. The CFA arthritis differs even more, as it involves not only myeloid but T cells as well. Furthermore, it is induced by the administration of exogenous mycobacterial antigens (the disease-inducing antigen being a bacterial heat shock protein cross-reactive with cartilage proteoglycan), thereby not resembling the autoinflammatory nature of RA^26^. However, similar to our findings, in the carrageenan-induced joint inflammation of the rat intraarticular administration of RTX or capsaicin not only induced a long-lasting analgesia, but also significantly reduced edema formation^27,28^.

Development of cold allodynia that remains persistent even after the decline of the acute inflammation was previously reported in the CAIA model, similarly to our results^29^. Furthermore, we observed a moderate, but significant facilitating effect of RTX-pretreatment on this phenomenon being more prominent in the early phase of the experiment. While seemingly perplexing, such diverging effects of systemic RTX-pretreatment on thermal and mechanical allodynia have been observed in previous studies, and were attributed mainly to the damage of myelinated afferent fibers, and consquent abnormal sprouting^30^. However, the possible confounding effect of behavioral changes due to RTX-pretreatment also cannot be ruled out.

While presence of thermal hyperalgesia in CAIA was shown by others by using the Hargreaves method^23^, our study using the increasing temperature hot-plate test could not corroborate this, and we did not find any significant difference between desensitized and non-pretreated animals. This is however, in agreement with our earlier results with the K/BxN serum-transfer arthritis, where the noxious heat threshold of the plantar surface of the paw was also unaffected by the inflammation of the joints^19^.

The moderate bone structural changes observed in our experiment reflect on the self-limiting and transient nature of the CAIA model. Naïve mice demonstrated a slight decrease of bone volume and increased trabecular separation, in good agreement with earlier results showing that CAIA is mainly characterized by trabecular bone loss due to increased osteoclast activity^31,32^. The increased bone surface porosity to the best of our knowledge has not been reported previously, however it is a logical consequence of increased bone resorption. RTX-pretreated mice display a slight increase of bone mass immediately upon the induction of arthritis, whilst formation of surface bone pores, a hallmark feature of arthritic bone resorption is diminished. Previously in the K/BxN serum-transfer model we have also observed a paradoxical bone neoformation in RTX desensitised animals, and also knockout mouse strains deficient in the neuropeptide pituitary adenylate-cyclase activating polypeptide (PACAP)^19,33^. The present results thus corroborate that defunctionalization of capsaicin-sensitive afferents leads to altered bone metabolic response during joint inflammation, shifting from bone resorption towards increased bone neoformation.

Histopathological examination of the tibiotarsal joint did not show any major bone and cartilage alterations neither in response to arthritis nor RTX desensitization in our model. These data clearly suggest that micro-CT is more sensitive and sophisticated technique to evaluate arthritis-related microarchitectural bone changes.

In summary, capsaicin sensitive afferents are pivotal factors in the development of autoantibody-induced joint inflammation, but the overall effect of their defunctionalization is greatly dependent on the pathophysiological mechanisms involved in the model. While their importance in facilitating inflammatory mechanical hyperalgesia has been supported by all prior experiments, their deactivation in the CAIA model leads to diminished arthritis severity and an absence of inflammatory bone loss. Thus, the importance of peptidergic sensory afferents in arthritis goes well beyond their already established importance in arthritic pain, representing a common denominator through which inflammatory and structural changes of RA could be influenced. The observed significant difference between different RA models highlights the importance of careful selection, and if possible cross-comparison of the different models. This is crucial for making conclusions about the role of sensory afferents and their mediators in immune-mediated arthritides. This is particularly important, as most models are narrowed down to only on a single signaling pathway, thereby not resembling the heterogenicity of the human disease.

## Methods

### Ethics

All experimental procedures were carried out according to the 1998/XXVIII Act of the Hungarian Parliament on Animal Protection and Consideration Decree of Scientific Procedures of Animal Experiments (243/1988) and complied with the recommendations of the International Association for the Study of Pain. The studies were approved by the Ethics Committee on Animal Research of University of Pécs according to the Ethical Codex of Animal Experiments and licence was given (licence No.: BA 02/2000-2/2012).

### Animals

Experiments were performed on 10–12-week-old male C57Bl/6 mice weighing 25–30 g. The original breeding pairs were purchased from Charles-River Hungary. The animals were bred and kept in the Laboratory Animal House of the Department of Pharmacology and Pharmacotherapy of the University of Pécs at 24–25 °C, provided with standard mouse chow and water *ad libitum* and maintained under a 12-h light-dark cycle.

### Resiniferatoxin pretreatment

Mice were pretreated with increasing doses (30, 70, 100 μg/kg s.c. on 3 consecutive days) of resiniferatoxin (RTX, Sigma-Aldrich), a potent TRPV1 receptor agonist, inducing a permanent depletion of capsaicin-sensitive afferents (desensitization or sensory neuronal deletion)^19,34,35^. To diminish the acute effects of RTX (bronchoconstriction and mucus secretion) the animals were simultaneously treated with a solution of 4% theophylline-ethylendiamine (Gedeon Richter Plc., Hungary), 4% terbutaline-sulphate (AstraZeneca Ltd., Hungary) and 2% atropine-sulphate (Egis Pharmaceuticals Plc., Hungary)^21^. The success of the desensitization was controlled by the lack of eye wiping response upon application of capsaicin eye drops (50 μl, 0.1%) 14 days thereafter^18^. Mice showing nocifensive response were excluded from the study.

### Induction of arthritis

The ArithroMab™ (MD Biosciences) antibody cocktail (4 mg/mouse) was injected into the tail vein two weeks after the desensitization under ketamine-xylazine anesthesia (ketamine 100 mg/kg; xylazine 5 mg/kg body weight i.p.). Lipopolysaccharide (100 μg/mouse) was i.p. injected three days thereafter. Sham-treated animals served as controls^36^.

### Arthritis severity and paw volume assessment

Hind paw volume was evaluated using plethysmometry (Ugo Basile 7140) at 2–3 days intervals, while clinical severity was semiquantitatively scored daily using a 0–10 grading system as described previously (0–0.5: normal, 10: maximal inflammation) based on the hallmark features of edema and hyperemia^37^.

### Evaluation of mechanical and thermal hyperalgesia and cold allodynia

The mechanonociceptive threshold of the hindlimb was assessed by dynamic plantar esthesiometry (Ugo Basile 37400), before and following arthritis induction at 2–3 days intervals. Mechanical hyperalgesia was expressed as % of initial control thresholds. Thermal hyperalgesia was evaluated using an increasing temperature hot plate (IITC Life Sciences). The platform of the device was heated until nocifensive reactions were observed (jumping, shaking, lifting or licking either limb), or until the preset maximum was attained (53 °C). The nociceptive threshold was expressed in °C^33^. Cold allodynia was determined by measuring the hind paw withdrawal latency from 0 °C water in seconds.

### Determination of joint function and body weight

To evaluate grasping ability the mice were placed onto a horizontal wire mesh grid which was turned over and maintained in this position for 30s or until the animal fell. This simple test has been previously successfully employed as a rapid screening tool for functional disability due to joint inflammation^37^. Body weight in grams was measured daily prior to all other tests and was expressed in % compared to an average of three initial self controls^38^.

### *In vivo* micro-computed tomography (micro-CT) of the ankle joint

The right tibiotarsal joints were imaged repeatedly (days 0, 7, 14, 21) by *in vivo* micro-CT (SkyScan 1176, Bruker) with a voxel size of 17.5 μm under ketamine-xylazine anesthesia as described earlier (average anesthesia duration: 30–40 min, scan duration 7–10 min). Bone structural changes were evaluated using the CT Analyser^®^ software by applying regions of interests (ROI) of identical size around the ankle joints^33^. ROIs were applied by first realigning the scanned distal part of the tibia so that it is perpendicular to the axial imaging plane, after which the last slice still including a part of the tibia was identified, and used as an anatomical reference point to establish a 3D geometric ROI. Within the ROIs the structural parameters such as bone volume (μm^3^), open pore volume (μm^3^) were expressed as percentage of the total ROI volume. Bone trabecularity was characterized by calculating average trabecular thickness and separation (μm).

### Histological sectioning and examination of the tibiotarsal joint

Tibiotarsal joints were obtained on day 21, at the end of the experiment, when the inflammatory phase is over as shown by our functional data and also the literature^29,39,40^. After fixation, decalcification and dehydration samples were embedded in paraffin, 3–5 µm sections were made and stained with hematoxylin-eosin^18^ in order to assess synovial proliferation, bone erosion and cartilage distruction and to compare the microscopical bone structure changes with the micro-CT data.

### Statistical analysis

Statistical evaluation was performed using GraphPad Prism^®^. All data points represent mean ± SEM. Functional and micro-CT results were assessed by two-way repeated measures ANOVA followed by Sidak’s post-test for multiple comparisons. Survival curves of grasping ability were analysed by the Mantel-Cox test. In all cases p < 0.05 was considered to be statistically significant.

## Electronic supplementary material

Supplementary Figure

## Data Availability

The datasets generated and analysed during the current study are available from the corresponding author on reasonable request.
