# Supplementary material for: Complex Role of Capsaicin-Sensitive Afferents in the Collagen Antibody-Induced Autoimmune Arthritis of the Mouse
Source: Sci Rep. 2018 Oct 29;8:15916. doi: 10.1038/s41598-018-34005-6 (PMC6206070; doi:10.1038/s41598-018-34005-6)

Supplementary material

Complex Role of Capsaicin-Sensitive Afferents in the Collagen Antibody-Induced Autoimmune Arthitis of the Mouse

Éva Borbély[1,2], Tamás Kiss[1], Krisztina Szabadfi[3†], Erika Pintér[1,2], János Szolcsányi[1,2], Zsuzsanna, Helyes[1,2*], Bálint Botz[1,4*]

[1]University of Pécs, Medical School, Department of Pharmacology and Pharmacotherapy, János Szentágothai Research Centre, Molecular Pharmacology Research Team & Centre for Neuroscience, Pécs, Hungary. [2]MTA-PTE Chronic Pain Research Group, Pécs, Hungary, [3]University of Pécs, Department of Experimental Zoology and Neurobiology, Pécs, Hungary. [4]University of Pécs, Medical School, Department of Radiology, Pécs, Hungary.

[†]Posthumous authorship.

*These authors made equal contribution to this work.

Address for correspondence and reprint requests to Zsuzsanna Helyes MD, PhD, DSc, Department of Pharmacology and Pharmacotherapy, University of Pécs, Medical School, Szigeti u. 12, 7624 Pécs, Hungary. E-mail: zsuzsanna.helyes@aok.pte.hu

Supplementary Figure Legend

Figure S1. Change of wire grid grasping ability expressed as percentage compared to pretreatment controls.

Figure S1.

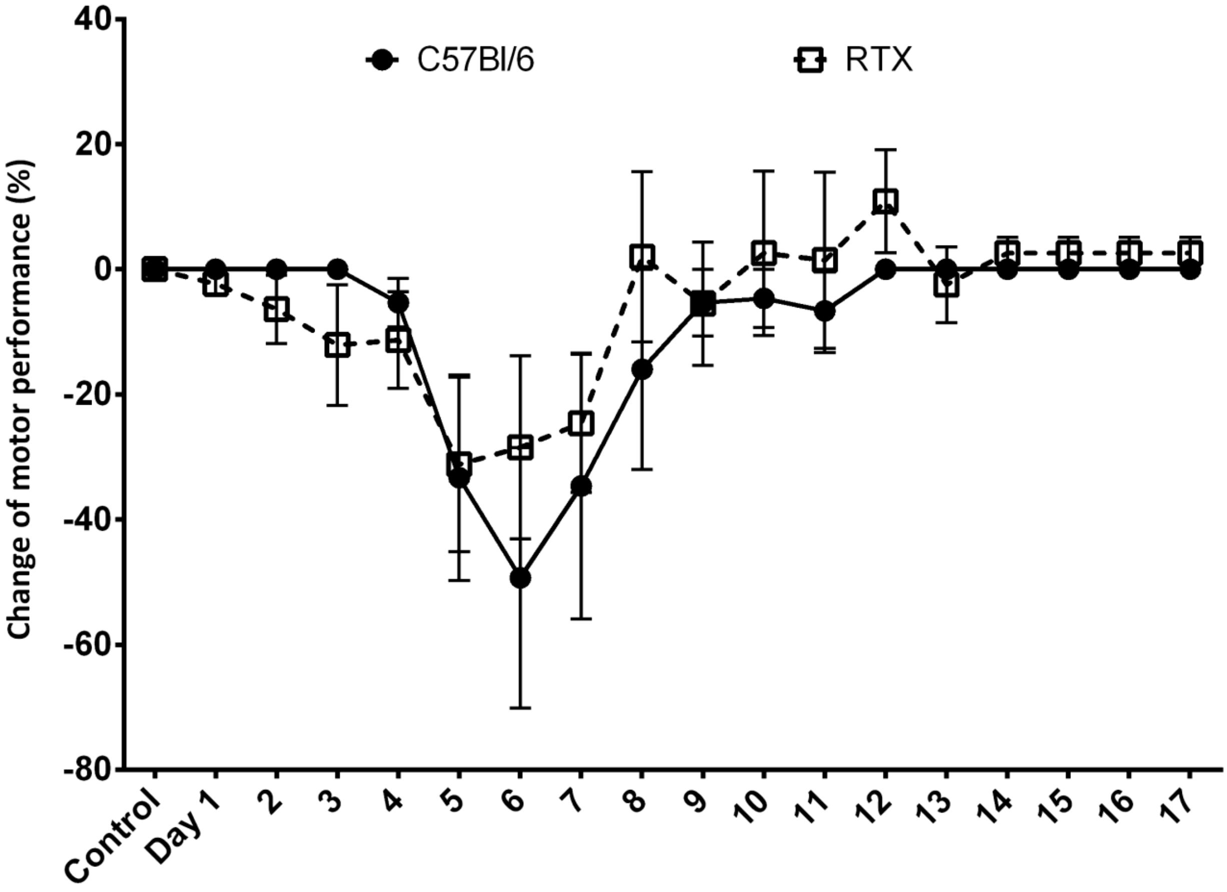

Supplement: Supplementary file 1 — Supplementary Figure [file 41598_2018_34005_MOESM1_ESM.pdf]
